# Chromosomal features of *Escherichia coli* serotype O2:K2, an avian pathogenic *E. coli*

**DOI:** 10.1186/s40793-017-0245-3

**Published:** 2017-05-10

**Authors:** Steffen L. Jørgensen, Egle Kudirkiene, Lili Li, Jens P. Christensen, John E. Olsen, Lisa Nolan, Rikke H. Olsen

**Affiliations:** 1 Department of Veterinary and Animal Sciences, Faculty of Health and Medical Sciences, University of Copenhagen, Stigboejlen 4, 1870 Frederiksberg C, Denmark; 2 College of Light Industry and Food Sciences, South China University of Technology, Guangdong, Zhongshan Rd, People’s Republic of China; 3 Department of Veterinary Microbiology and Preventive Medicine, College of Veterinary Medicine, Iowa State University, 1802 Elwood Drive, VMRI #2, Ames, IA 50011 USA

**Keywords:** Avian pathogenic *Escherichia coli*, Genome sequencing, Chromosome, Colibacillosis, Chicken

## Abstract

*Escherichia coli* causing infection outside the gastrointestinal system are referred to as extra-intestinal pathogenic *E. coli.* Avian pathogenic *E. coli* is a subgroup of extra-intestinal pathogenic *E. coli* and infections due to avian pathogenic *E. coli* have major impact on poultry production economy and welfare worldwide. An almost defining characteristic of avian pathogenic *E. coli* is the carriage of plasmids, which may encode virulence factors and antibiotic resistance determinates. For the same reason, plasmids of avian pathogenic *E. coli* have been intensively studied. However, genes encoded by the chromosome may also be important for disease manifestation and antimicrobial resistance. For the *E. coli* strain APEC_O2 the plasmids have been sequenced and analyzed in several studies, and *E. coli* APEC_O2 may therefore serve as a reference strain in future studies. Here we describe the chromosomal features of *E. coli* APEC_O2. *E. coli* APEC_O2 is a sequence type ST135, has a chromosome of 4,908,820 bp (plasmid removed), comprising 4672 protein-coding genes, 110 RNA genes, and 156 pseudogenes, with an average G + C content of 50.69%. We identified 82 insertion sequences as well as 4672 protein coding sequences, 12 predicated genomic islands, three prophage-related sequences, and two clustered regularly interspaced short palindromic repeats regions on the chromosome, suggesting the possible occurrence of horizontal gene transfer in this strain. The wildtype strain of *E. coli* APEC_O2 is resistant towards multiple antimicrobials, however, no (complete) antibiotic resistance genes were present on the chromosome, but a number of genes associated with extra-intestinal disease were identified. Together, the information provided here on *E. coli* APEC_O2 will assist in future studies of avian pathogenic *E. coli* strains, in particular regarding strain of *E. coli* APEC_O2, and aid in the general understanding of the pathogenesis of avian pathogenic *E. coli*.

## Introduction

Avian pathogenic *Escherichia coli* strains are the etiological agent of colibacillosis in birds, which is one of the most significant infectious diseases affecting poultry [6, 33]. In the veterinary field, avian pathogenic *E. coli* associated diseases implies economic losses in the poultry industry worldwide [27]. Furthermore, avian pathogenic *E. coli* strains have been reported to represent a zoonotic risk, as the population of avian pathogenic *E. coli* shares major genomic similarities with the population of human uropathogenic *E. coli* [22, 44]. Despite importance of this disease, the importance of the genetic features and genome diversity with avian pathogenic *E. coli* remains to be fully understood. Here we report the full genome sequence and sequence annotation of *E. coli* APEC_O2. *E. coli* APEC_O2 is an *E coli* strain (serotype O2:K2) isolated from the joint of a chicken in 2014 [22]. *E. coli* APEC_ O2 possesses two large, well-characterized plasmids [22, 23] which have been used in antimicrobial and virulence studies [21, 36], while no characterization of the chromosomal features have been available until now.

## Organism information

### Classification and features

**Fig. 1:**
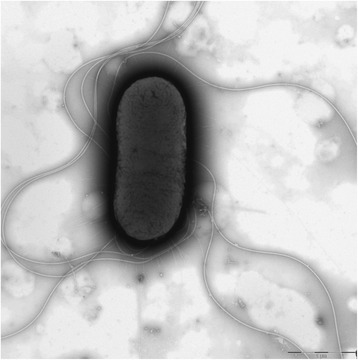
Transmission electron micrograph of APEC_O2. The strain is a short to medium rod-shaped bacterium with a length of 1–2 μm. It moves via peritrichous flagella. The magnification rate is 20,000×. The scale bar indicates 1 μm

*E. coli* is a Gram-negative, non-spore forming, rod-shaped bacteria belonging to the *Enterobacteriaceae* family [34]. *E. coli* APEC_O2 is motile by the means of peritrichous flagella (Fig. 1), is non-pigmented, oxidase-negative, facultative anaerobe and is growing with a optimum between 37 and 42 °C. *E. coli* APEC_O2 is positive for indole production, nitrate reduction, and urease but is hydrogen-sulfide negative. The strain is positive for lysine-decarboxylase and ornithine-decarboxylase activity, and produce acid and gas while fermenting d-glucose. *E. coli* APEC_O2 fermented d-trehalose, d-sorbitol, d-mannitol, l-rhamnose, d-glucose, d-maltose, and d-arabinose, but does grown on citric acid, inositol or gelatin. Furthermore, *the strain* does not produce acetoin (Voges–Proskauer negative), and does not utilize malonate.

The primary habitat of *E. coli* is in the gastrointestinal tract (GIT) of humans, many of the warm blooded animals as well as poultry [24]. Most strains of *E. coli* are considered commensal strains of the GIT, however, certain pathovars of *E. coli* may cause intestinal disease, while other cause disease when entering the extra-intestinal compartments of the body [30]. Avian pathogenic *E. coli* is an important agent of extra-intestinal diseases in poultry, including respiratory, hematogenous, ascending and skin infections, collectively called colibacillosis [33]. *E. coli* APEC_O2 was obtained from a joint of chicken with arthritis in 2014 (Table 1), and has subsequently been used in different scientific studies [22, 23, 36]. The serotype of *E. coli* APEC_O2 is O2:K2 [22], which is one of the most common serotypes among avian pathogenic *Escherichia coli* worldwide [33].

**Table 1:** Classification and general features of the *E. coli* APEC_ O2 strain

MIGS ID	Property	Term	Evidence code^a^
	Classification	Domain *Bacteria*	TAS [41]
		Phylum *Proteobacteria*	TAS [16]
		Class *Gammaproteobacteria*	TAS [40]
		Order’ *Enterobacteriales”*	TAS [16, 40]
		Family *Enterobacteriaceae*	TAS [8]
		Genus *Escherichia*	TAS [13]
		Species *Escherichia coli*	TAS [13]
	Gram stain	Negative	TAS [39]
	Cell shape	Rod	TAS [39]
	Motility	Motile	TAS [39]
	Sporulation	None-sporeforming	TAS [39]
	Temperature range	Mesophile	TAS [39]
	Optimum temperature	37 °C	TAS [39]
	pH range; Optimum	5.5–8.0; 7.0	TAS [39]
	Carbon source	Carbohdrates, salicin, sorbitol, mannitol, indole, peptides	TAS [39]
MIGS-6	Habitat	Host-associated	TAS [14]
MIGS-6.3	Salinity	Not reported	
MIGS-22	Oxygen requirement	Aerobe and facultative anaerobe	TAS [39]
MIGS-15	Biotic relationship	Parasitism	TAS [6, 14]
MIGS-14	Pathogenicity	Pathogenic	TAS [6, 14]
MIGS-4	Geographic location	USA	NAS
MIGS-5	Sample collection	2014	
MIGS-4.1	Latitude	Not reported	
MIGS-4.2	Longitude	Not reported	
MIGS-4.4	Altitude	Not reported	

^a^ Evidence codes - TAS: Traceable Author Statement (i.e., a direct report exists in the literature); NAS: Non-traceable Author Statement (i.e., not directly observed for the living, isolated sample, but based on a generally accepted property for the species, or anecdotal evidence). These evidence codes are from the Gene Ontology project [2]

**Fig. 2:**
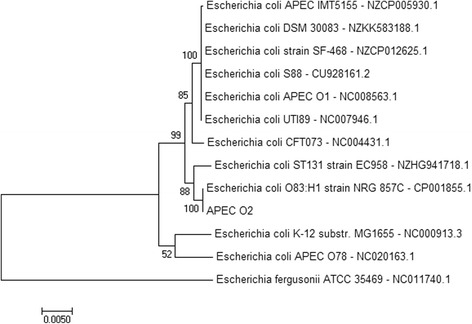
Maximum likehood tree of APEC_O2 relative to other closely related strains. The phylogenetic tree was constructed from the concatenated seven housekeeping genes (*adk, fumC, gyrB, icd, mdh, purA,* and *recA*) in MEGA software version 7. *Escherichia fergusonii* (ATCC35469) was used as an out-group. Bootstrap values of 500 replicates are indicated at the nodes. The scale bar indicates nucleotide diversity between the strains

A Maximum Likelihood method phylogenetic tree based on the concatenated seven housekeeping genes of *E. coli*, were made in MEGA (version 7) [37], with 500 bootstrap (Fig. 2). Housekeeping gene sequences from the following strains were used to construct the phylogenetic tree: *E. coli* str. K-12 str. MG1655, NC_000913.3, *E. coli* APEC O1, NC_008563.1, *E. coli* UTI89, NC_007946.1, *E. coli* S88, CU928161.2, *E. coli* CFT073, NC_004431.1, *E. coli* APEC O78, NC_020163.1, *E. coli* ST131 strain EC958, Z_HG941718.1, *E. coli* strain SF-468, NZ_CP012625.1, *E. coli* APEC IMT5155, NZ_CP005930.1, *E. coli* O83:H1 str. NRG 857C, CP001855.1, *E. coli* DSM 30083, NZ_KK583188.1, and *Escherichia fergusonii* ATCC 35469, NC_011740.1.

Two large plasmids of APEC_O2 (pAPEC-O2-ColV and pAPEC-O2-R) have previously been described in details [22, 23]. Various antibiotic resistance and virulence associated genes of APEC_O2 have been identified on these two plasmids. The plasmid pAPEC-O2-ColV has been reported to be co-transferred with plasmid pAPEC-O2-R into the non-virulent *E. coli* DH5α strain, resulting in an increase in antibiotic resistance and virulence of the recipient strain [21].

## Genome sequencing information

### Genome project history

The strain of *E. coli* APEC_O2 was selected for whole genome sequencing at the Department of Veterinary Disease Biology, Denmark, because information regarding the chromosomal background of the strains was lacking. Sequence assembly and annotation were completed in December 2015, and the draft genome sequence was deposited in GenBank under accession number LSZR00000000. A summary of the project information and its association with “Minimum Information about a Genome Sequence” according to Field et al. [15] is provided in Table 2.

**Table 2:** Project information

MIGS ID	Property	Term
MIGS 31	Finishing quality	Drafted
MIGS-28	Libraries used	Paired-end Nextera XT DNA
MIGS 29	Sequencing platforms	Illumina MiSeq
MIGS 31.2	Fold coverage	33.0x
MIGS 30	Assemblers	CLC NGS Cell v. 7.0.4
MIGS 32	Gene calling method	GeneMarkS+
	Locus Tag	AZE29
	Genbank ID	LSZR00000000
	GenBank Date of Release	2016/04/14
	BIOPROJECT	PRJNA312653
	BioSample Accession	SAMN04503534
MIGS 13	Source Material Identifier	APEC_O2
	Project relevance	Pathogenic bacterium, biotechnological

### Growth conditions and genomic DNA preparation

One colony of *E. coli* APEC_O2 cultured on agar plates (Blood agar base, Oxoid, Roskilde, Denmark), supplement with 5% bovine blood was inoculated in 10 mL Brain and Heart Infusion (BHI) broth for 18 h yielding a final density of 10^9^ colony forming units per mL BHI broth. DNA from 1 mL of the APEC_O2 inoculated was extracted using DNeasy Blood & Tissue Kit (*Qiagen*, USA). The quantity (127 ng/μl) and quality of DNA (ratio of light absorption at wavelengths 260/280 was 1.81 and 1.99 at wavelengths 260/230) was assessed using Nanodrop (Thermo Scientific, USA).

### Genome sequencing and assembly

Genome sequencing was performed using the MiSeq instrument (Illumina) at a 300-bp paired-end-read format. CLC Genomic Workbench 6.5.1 software package (CLC, Denmark) was used to perform adapter trimming and quality assessment of the reads. Sequencing reads were *de novo* assembled using the SPAdes v.3.5.0 [5]. The quality of the assembly was evaluated with QUAST v.2.3 [18]. The run yielded 981,795 high quality filtered reads containing 5,166,016 bases, which provided an average of 33-fold coverage of the genome. The assembly resulted in 304 contigs ranging from 216 to 192,013 bp in size. The contigs were aligned with two previously published *E. coli* APEC_O2 plasmids ColV and R (R) using the progressive Mauve algorithm in Mauve 2.3.1 [11], and those corresponding to the plasmid sequences were removed. The final *E. coli* APEC_O2 chromosomal genome had the size of 4.9 Mbp, and was assembled into 261 contigs. The relative large number of contigs is most likely due to a high number of mobile elements found in draft genome of *E. coli* APEC_O2 (please see result section). Genes in internal clusters were detected using CD-HIT v4.6 with thresholds of 50% covered length and 50% sequence identity [9].

### Genome annotation

The draft genome sequence of *E. coli* APEC_O2 was analyzed using Glimmer 3.0 and GeneMark for gene prediction [7, 12, 25]. Ribosomal RNA identification was performed using RNAmmer 1.2 [26]. The predicted protein coding sequences were annotated and protein features were predicted by BASys analysis using the NCBI database [38].

## Genome properties

The complete draft genome of *E. coli* APEC_O2 consists of one circular chromosome of 4,908,820 bp with an average G + C content is 50.69%. In addition *E. coli* APEC_O2 contains two plasmids: pAPEC-O2-ColV and pAPEC-O2-R, which are not included in the analysis or features descripted in the present study (Table 3). In total, 4938 genes were predicted on the chromosomal genome, of which 110 coded for RNA related genes, 4672 were protein coding genes, and 156 were pseudogenes (Table 4). In total, 4099 genes were assigned in COG functional categories and listed in Table 5.

**Table 3:** Summary of APEC_O2 genome: one chromosome and two plasmids

Label	Size (Mb)	Topology	INSDC identifier	RefSeq ID
Chromosome	4,908,820	Circular	GenBank	GCA_001620375.1
pAPEC-O2-ColV	0.18	Circular	GenBank	AY545598.5
pAPEC-O2-R	0.1	Circular	GenBank	AY214164.3

**Table 4:** Genome statistics

Attribute	Value	% of Total
Genome size (bp)	4,908,820	100.00
DNA coding (bp)	4,320,149	88.01
DNA G + C (bp)	2,488,281	50.69
DNA scaffold	261	-
Total genes	4938	100
Protein coding genes	4672	94.61
RNA genes	110	2.22
Pseudo genes	156	3.16
Genes in internal clusters	252	5.1
Genes with function prediction	4209	85.24
Genes assigned to COGs	4099	83.00
Genes with Pfam domains	4713	95.44
Genes with signal peptides	550	11.14
Genes with transmembrane helices	1107	22.42
CRISPR repeats	2

**Table 5:** Number of genes associated with general COG functional categories

Code	Value	% age	Description
J	200	4.06	Translation, ribosomal structure and biogenesis
A	0	0.00	RNA processing and modification
K	319	6.47	Transcription
L	231	4.67	Replication, recombination and repair
B	0	0.00	Chromatin structure and dynamics
D	35	0.71	Cell cycle control, Cell division, chromosome partitioning
V	0	0.00	Defense mechanisms
T	161	3.26	Signal transduction mechanisms
M	270	5.47	Cell wall/membrane biogenesis
N	143	2.89	Cell motility
U	0	0.00	Intracellular trafficking and secretion
O	163	3.31	Posttranslational modification, protein turnover, chaperones
C	327	6.61	Energy production and conversion
G	471	9.53	Carbohydrate transport and metabolism
E	384	7.78	Amino acid transport and metabolism
F	109	2.21	Nucleotide transport and metabolism
H	156	3.16	Coenzyme transport and metabolism
I	119	2.41	Lipid transport and metabolism
P	221	4.47	Inorganic ion transport and metabolism
Q	61	1.24	Secondary metabolites biosynthesis, transport and catabolism
R	393	7.95	General function prediction only
S	336	6.81	Function unknown
-	734	14.86	Not in COGs

The total is based on the total number of protein coding genes in the genome

MLST finder 1.8 [28] was used to identify the sequence type of *E. coli* APEC_O2 as ST135, while SeroTypeFinder [20] was used to confirm the serotype of *E. coli* APEC_O2 as O2:K2 as published by others [22].

VirulenceFinder 1.5 and ResFinder 2.1 were used for identification of intrinsic genes associated with virulence and antibiotic resistance, respectively [19, 42]. Clustered regularly interspaced short palindromic repeat sequences were detected using CRISPR-finder [17]. IS-finder and PHAST were used for identification and location of insertion sequences and phages [35, 43].

**Fig. 3:**
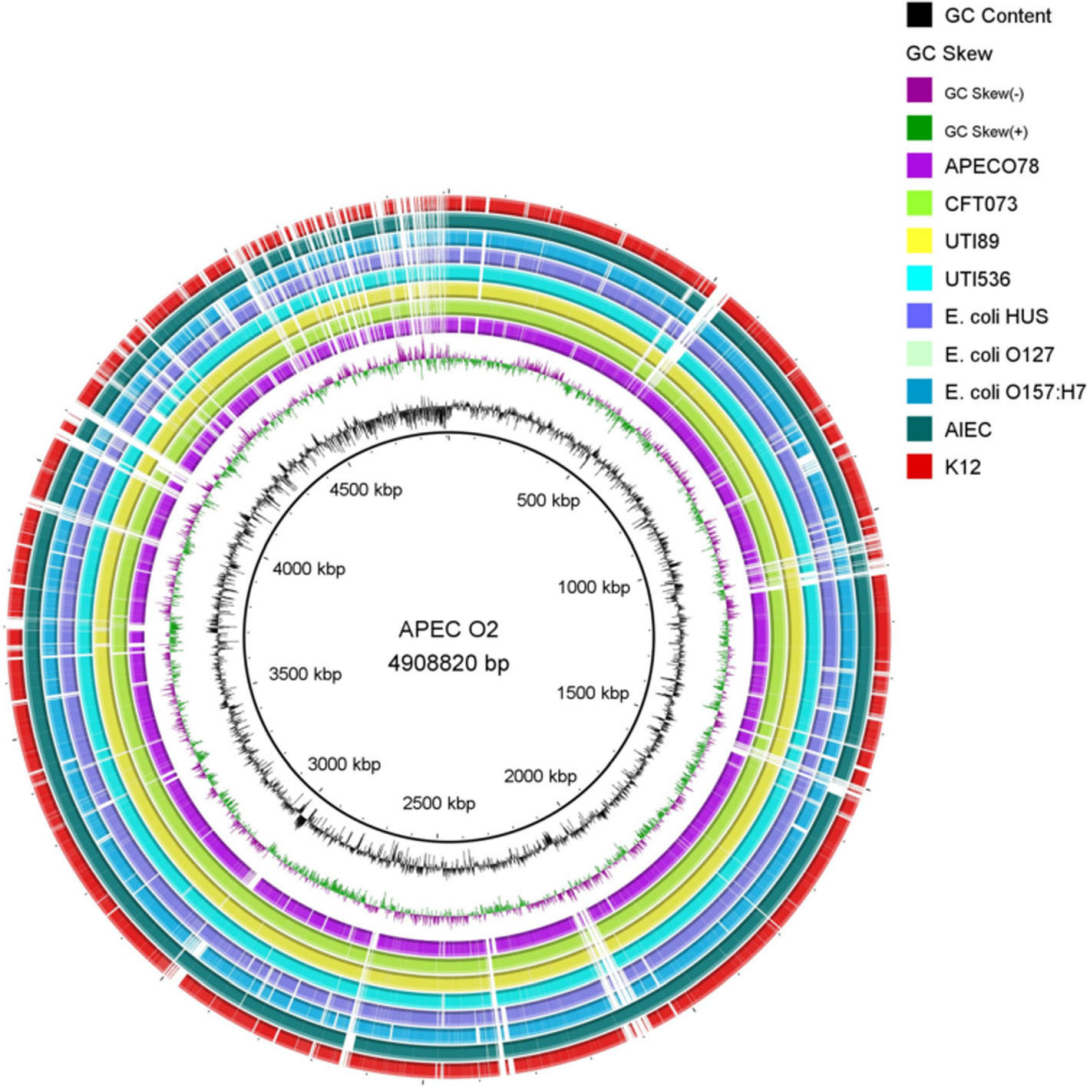
Genomic comparison of APEC_O2 with other strains of *Escherichia coli*. Genome wide comparison of APEC_O2 with the complete genomes of another Avian pathogenic *E. coli,* APECO78 (CP004009.1), three isolates of human urinary pathogenic *E. coli* (CFT073(NC_004431.1), UTI89 (NC_007946.1) and UTI536 (NC_008253.01), three isolates of intestinal pathogenic *E. coli* (*E. coli* HUS (PRJNA68275), *E. coli* O127 (PRJNA204937), *E. coli* O157:H7 (GCA_000008865.1) and AIEC (GCA_000183345.1), respectively) and a non-pathogenic *E. coli* (*E. coli*_K12 (GCA_000005845.2). Solid color of concentric rings indicated genomic areas also present in APEC_O2 (inner black circle), whereas absence of color in a ring indicates absence of the region

BLAST ring image generator (BRIG) [1] was applied to the compare the genome of *E. coli* APEC_O2 with APEC O78 (CP004009.1), three isolates of human urinary pathogenic *E. coli* isolates (CFT073 (NC_004431.1), UTI89 (NC_007946.1) and UTI536 (NC_008253.01)), three intestinal pathogenic *E. coli* (*E. coli* HUS (PRJNA68275), *E. coli* O127 (PRJNA204937), *E. coli* _O157:H7 (GCA_000008865.1) and AIEC (GCA_000183345.1), a non-pathogenic *E. coli* (*E. coli*_K12 (GCA_000005845.2) (Fig. 3).

**Fig. 4:**
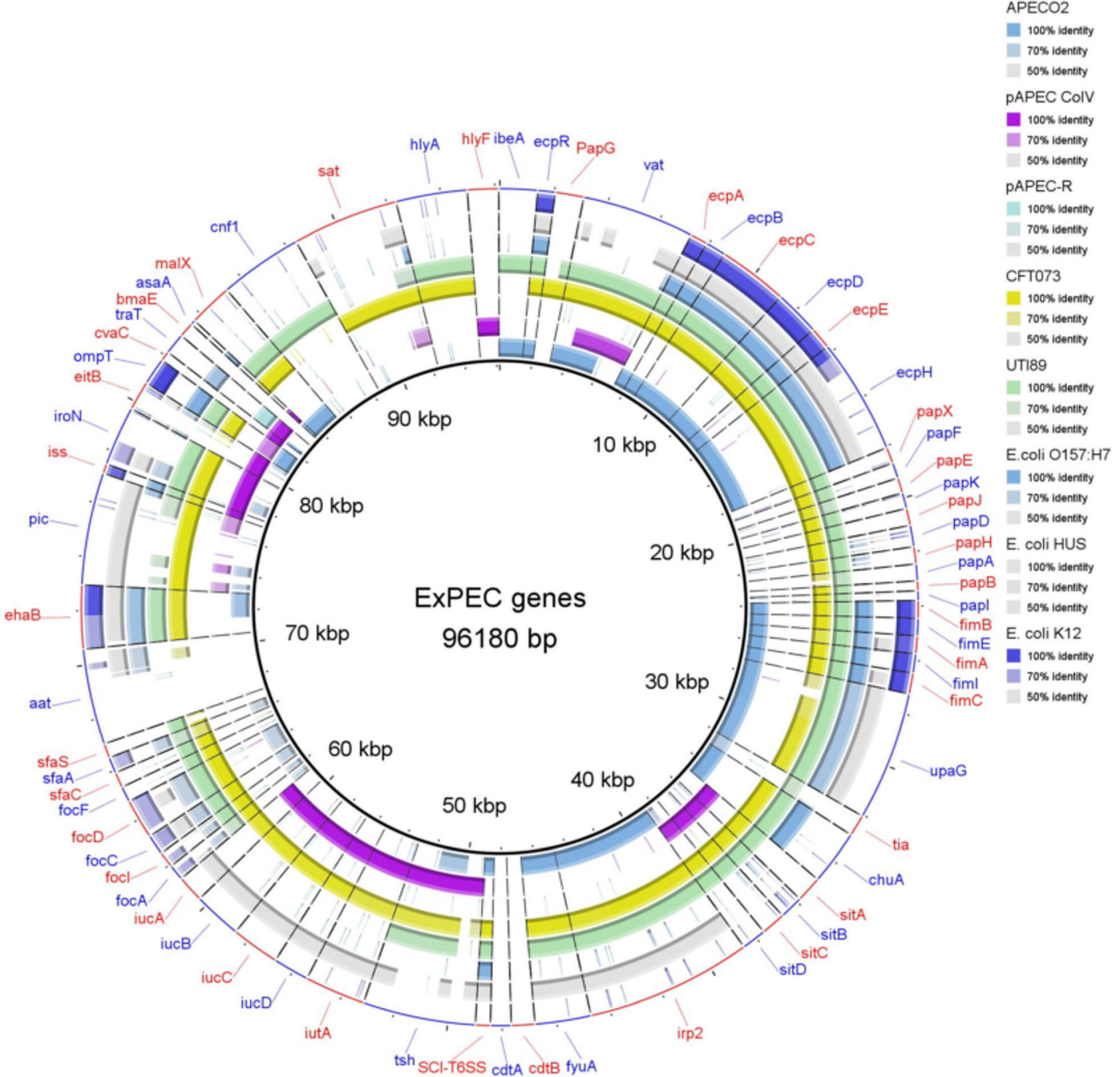
Screening for the presence of selected virulence genes. The presence or absence of 65 genes related to extra-intestinal disease in APEC_O2. For comparison reasons the genomes of two plasmids of the wildtype of APEC_O2 (pAPEC ColV(NC_007675.1) and APEC-R (AY214164)), in addition to the genomes of CFT073(NC_004431.1, UTI89 (NC_007946.1), *E. coli* HUS (PRJNA68275), *E. coli* O127 (PRJNA204937), *E. coli* O157:H7 (GCA_000008865.1) and *E. coli*_K12 (GCA_000005845.2), respectively, were also included in the analysis. All genomic sequences of the virulence genes were obtained from the online Virulence Factor Database (http://www.mgc.ac.cn/VFs/main.htm)

BRIG was also used to examine the genome of *E. coli* APEC_O2 for the presence of selected virulence genes. The sequences of sixty-two genes related to extra-intestinal virulence were extracted from the Virulence Factor Database [10] and blasted against the genome of *E. coli* APEC_O2. The virulence genes included six adhesins (*bma*, *ecp, pap, fim, foc,* and *sfa*), five toxins (*astA*, *cnf1*, *vat, cdt,, hlyF*), six auto-transporters (*aat, ehaB, pic, upaG*, *tsh,sat*), two invasion genes (*ibeA*, *tia*), 14 iron acquisition genes (*chuA*, *eitB, sitA, sitB, sitC, irp2, fyuA, ompT,iroN, iutA, iucA, iucB, iucC, iucD*), one gene of the type VI secretion system (*T6SS*) and four miscellaneous genes (*iss*, *cvaC, traT, malX)* (Fig. 4). The RAST server [4] was used to identify subsystem features in *E. coli* APEC_O2 and the type strain of *E. coli* (*E. coli* DMS 30038). *In silico* DNA-DNA hybridization (dDDH)similarities between the *E. coli* APEC_ O2 strain and the 12 strains used for the Maximum likelihood analysis, were calculated using the Genome-to-Genome Distance Calculator v. 2.1 [3].

### Insights from the genome sequence

Here we present the draft genome sequencing and annotation of the chromosome of the *E. coli* strain APEC_O2. Four thousand six hundred seventy two protein-coding sequences accounting for 94.61% of the total number of 4938 genes identified. This analysis predicted 82 insertion sequences and three phage associated sequences.

*E. coli* APEC_O2 was interestingly found to belong to sequence type ST135, which previously only sparsely have been associated with pathogenicity [32].

*E. coli* APEC_O2 is phylogenetically closely related to *E. coli* strain EC958, belonging to ST131, which is recognized as a leading contributor to human urinary tract infections, and to an adherent invasive *E. coli* strain (NRG EC958), which originally were isolated from a terminal patient suffering from Chron’s disease. The latter was quite unexpected, as intestinal and extra-intestinal pathogenic *E. coli* are believed to constitute two different pathotypes [24], however, other studies have suggested that there might be a phylogenetic relationship between adherent invasive *E. coli* and extra-intestinal pathogenic *E. coli* [29]. Adding to the suggested close relationship between adherent invasive *E. coli* and extra-intestinal pathogenic *E. coli*, in this case *E. coli* APEC_O2, was the finding of a dDDH estimate of 96.50% between the two strains, which is higher than the similarities to any of the other strains included in the phylogenetic analysis (Fig. 1, Table 6). Moreover, the similarity to *E. coli* strain EC958 were almost 10% lower, and the probability that *E. coli* APEC_O2 belong to the same subspecies (estimated by dDDH > 79%) were below 60%. (Table 6).

**Table 6:** DNA:DNA-hybridization (dDDH) of APEC_O2 to selected *E. coli* strains

	DDH estimate (GLM-based)	Probability that DDH > 70%	Probability that DDH > 79%
APEC_O2 versus:			
*E. coli* 1655 (NZCP005930.1)	74.80% [71.8–77.6%]	85.53%	37.84%
APEC01 (NC008563.1)	90.60% [88.3–92.4%]	95.98%	66.14%
*E. coli* APECO78 (NC020163.1)	74.70% [71.6–77.5%]	85.33%	37.53%
*E. coli* CFT073 (NC004431.1)	91.00% [88.8–92.8%]	96.13%	66.89%
*E.coli*_ST131_strain_EC958 (NZHG941718.1)	86.60% [84–88.8%]	94.48%	59.67%
*E.coli*_O83H1_strain_NRG_857C (CP001855.1)	96.50% [95.3–97.5%]	95.55%	74.94%
*E. fergusonii* ATCC 35469 (NC011740.1)^a^	40.30% [37.8–42.8%]	2.9%	0.73%
*E. coli* IMT5155 (NZCP005930.1)	90.90% [88.7–92.7%]	96.1%	66.7%
*E. coli* S88 (CU928161.2)	89.90% [87.6–91.8%]	95.77%	65.12%
*E. coli* SF/468 (NZCP012625.1)	90.50% [88.2–92.3%]	95.95%	65.89%
*E. coli* DMS 30083 (NZKK583188.1)	90.30% [88–92.2%]	95.89%	65.72%
*E. coli* UTI89 (NC004431.1)	91.10% [89–92.9%]	96.17%	67.05%

^*a*^ *E. fergusonii* ATCC 35469 (NC011740.1) was included to represent an out-group strain

**Fig. 5:**
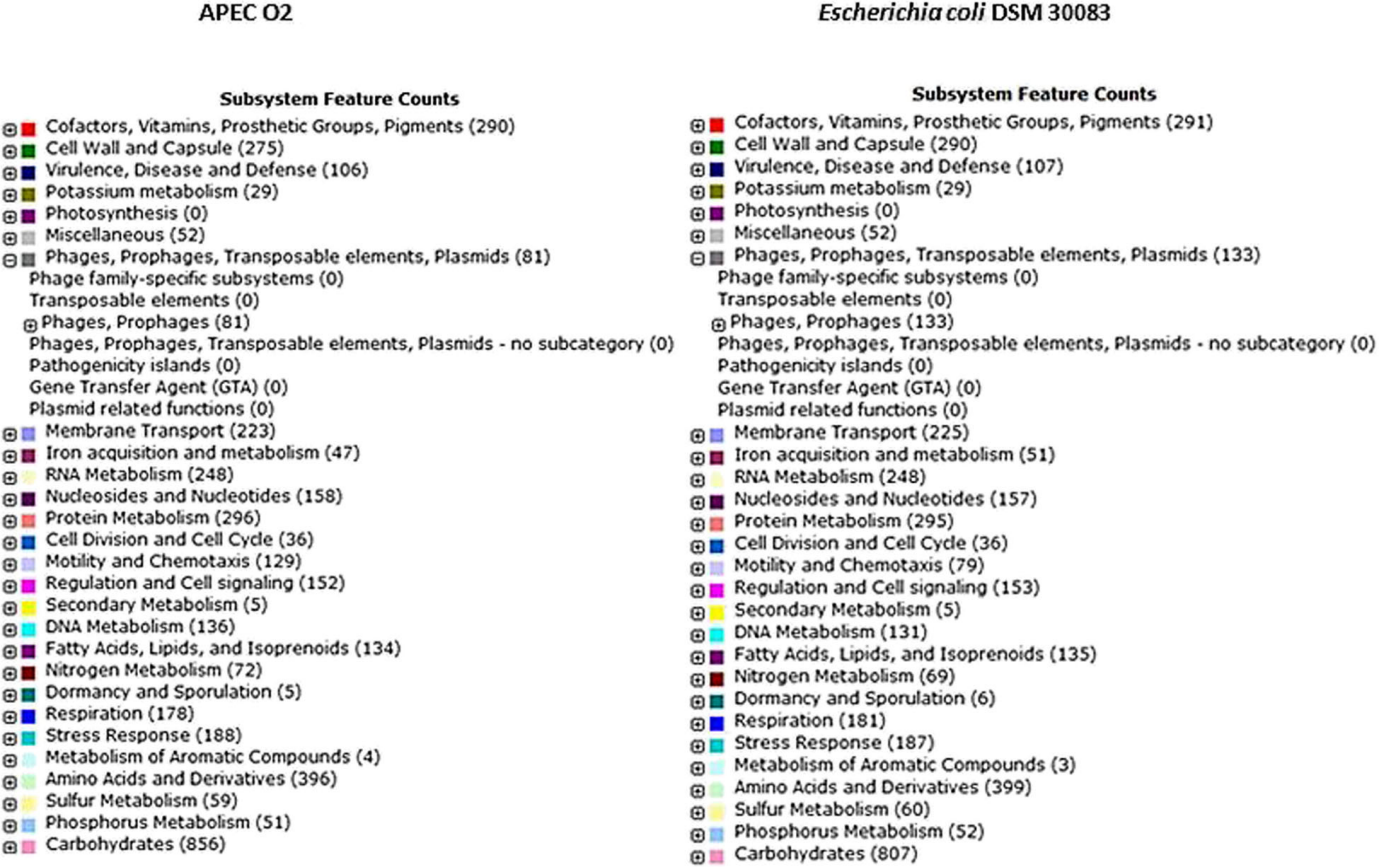
Subsystem feature counts in APEC_O2 and *E. coli* DMS 30083 (NZKK583188.1)

For comparison, the dDDH estimate between the type strain of *E. coli* (*E. coli* DSM) [31] and avian pathogenic *E. coli* were around 90%. The differences might be due to the considerably higher numbers of phage- and prophage regions in the type strain compared to *E. coli* APEC_O2 (Fig. 5). Besides difference in this feature, distribution of subsystem feature counts was highly similar between the two strains.

## Conclusions

In this study, we present the draft genome sequence of the chicken-derived *E. coli* isolate APEC_O2. The genome of *E. coli* APEC_O2 consists of a 4,908,820 bp long chromosome, containing 4672 protein coding genes. *E. coli* APEC_O2 furthermore contains two transferable plasmids, which carry several virulence and antibiotic resistance genes.

Previous studies have demonstrated close genetic resemblance between avian pathogenic *E. coli* and extra-intestinal pathogenic *E. coli* strains, and suggested poultry as a reservoir of extra-intestinal pathogenic *E. coli* strains associated with disease in humans, and as a possible route of transmission. In the present study full genomic comparison of genomes did not reveal closer genomic relationship between *E. coli* APEC_O2 and human extra-intestinal pathogenic *E. coli* strains than to human *E. coli* strains of other pathotypes similarities. Nevertheless, the chromosomal contents of APEC_O2 did harbor genes of importance for extra-intestinal disease. In addition, dDDH similarities indicated that APEC_O2 had equally high similarity to strains uropathogenic strains as to other avian pathogenic *E. coli* strain and the type strain of *E. coli* *.*

More surprising, *E. coli* APEC_O2 had the highest dDDH similarity to an adherent invasive *E. coli*, as intestinal *E. coli* original were considered to constitute a pathotype very different from extra-intestinal pathogenic *E. coli*.

Conclusively, the draft genome sequence and annotation of the pathogenic avian pathogenic *E. coli* strain APEC_O2 provides new information, which may add for future studies of the pathogenesis, transmission and zoonotic risk related to avian pathogenic *E. coli*.

## Abbreviations

BHI: Brain and Heart Infusion
BRIG: BLAST Ring Image Generator
CRISPR: Clustered Regularly Interspaced Short Palindromic Repeats
dDDH: DNA-DNA hybridization
*E. coli*: *Escherichia coli*
GIT: Gastrointestinal tract
IS: Insertion sequences
MLST: Multi Locus Sequence typing
PHAST: PHAge Search Tool
ST: Sequence type
